# Deep Tracking Neck Abscesses, Mediastinitis, and Deep Vein Thrombosis Caused by *Aggregatibacter actinomycetemcomitans*

**DOI:** 10.1002/ccr3.72212

**Published:** 2026-03-09

**Authors:** Joshua Sia, Carly Hughes

**Affiliations:** ^1^ Department of Infectious Diseases Townsville University Hospital Australia; ^2^ James Cook University Australia

**Keywords:** abscess, *Aggregatibacter actinomycetemcomitans*, communicable disease, gram‐negative bacterial infections, mediastinitis

## Abstract

This case report describes a novel presentation of *Aggregatibacter actinomycetemcomitans* causing deep neck abscess, mediastinitis, and deep vein thrombosis. Clinicians should be aware that this organism can cause invasive extra‐oral infection. More research is required to define optimal treatment duration; however, our case demonstrates success with 6 weeks of antibiotics.

## 1 Introduction

Deep neck space infections with associated descending mediastinitis are serious infections, usually caused by polymicrobial odontogenic bacteria [1]. A case caused by *A. actinomycetemcomitans* has not been described. *A. actinomycetemcomitans* is a fastidious, facultative anaerobic, small gram‐negative rod that is considered normal flora of the human oral cavity. It frequently causes endogenous infection and is a major cause of periodontitis. Extra‐oral infections are rare [2]. Here we report a novel case of deep‐tracking neck abscess, mediastinitis, and deep vein thrombosis due to *A. actinomycetemcomitans* diagnosed using 16S ribosomal RNA (rRNA) sequencing of neck abscess tissue collected during surgery.

## 2 Case History/Examination

An Australian man in his 40s presented to a North Queensland Hospital with a one‐week history of left‐sided neck, soft tissue swelling and pain. This later developed into a purulent discharge over the previous 3 days. He reported associated fevers, dizziness, and malaise, which had worsened over the one‐week period. He was an active smoker with a 25‐pack‐year history and denied any alcohol or recreational drug use. He had no other significant past medical history. His initial observations showed a temperature of 36.2°C, heart rate of 99 beats per minute, respiratory rate of 18 breaths per minute and blood pressure of 120/79 mmHg. On physical examination, he was found to have two areas of soft tissue swelling measuring 5 × 3cm and 4 × 2cm, respectively, on the left neck. There was tenderness, fluctuance, and expressible purulent material on palpation. Poor oral hygiene and gingival retraction were noted. He had no lymphadenopathy and an unremarkable cardiovascular and respiratory examination.

## 3 Methods

**FIGURE 1:**
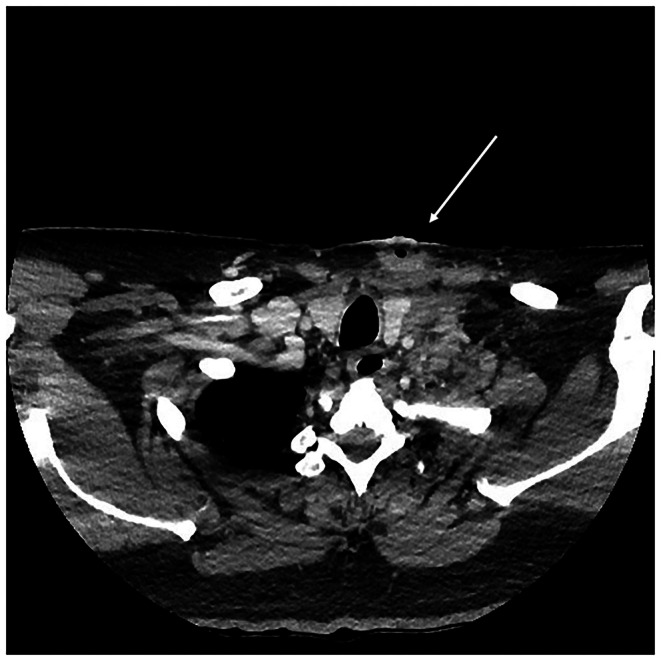
Subcutaneous abscesses in the left anterior and lateral neck tracking toward the carotid sheath and extending into the superior mediastinum with mediastinitis.

**FIGURE 2:**
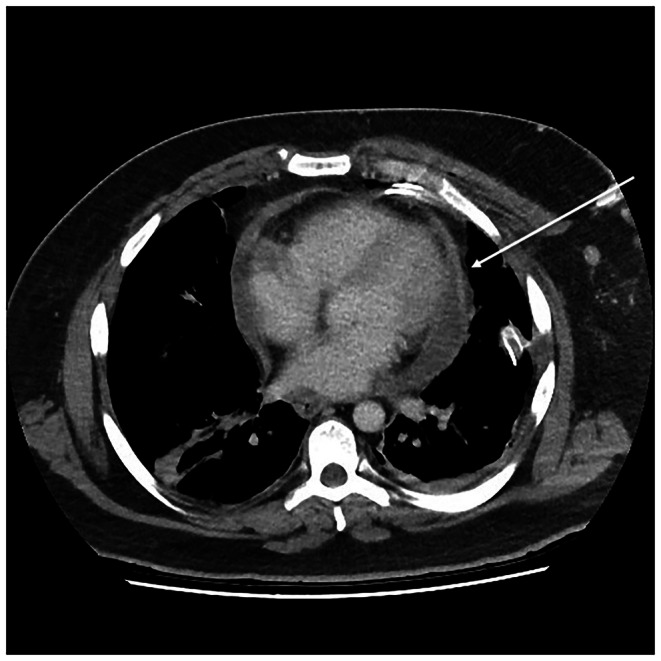
Mediastinitis with pericardial effusion.

**FIGURE 3:**
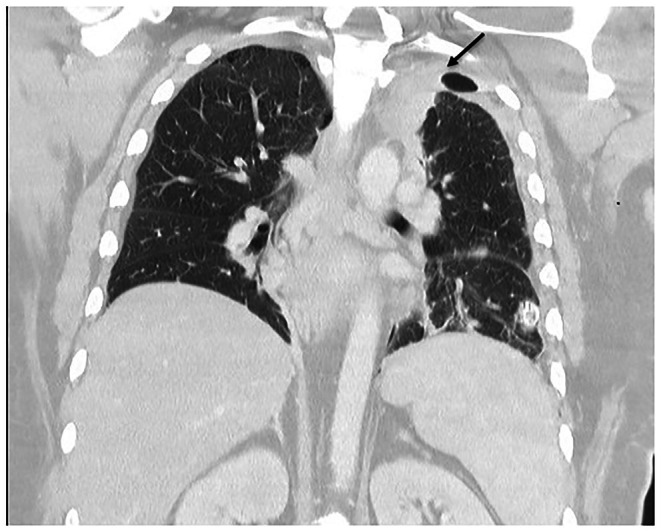
Left upper lobe consolidation.

Laboratory investigations showed an elevated C‐reactive protein (181 mg/L, RR ≤ 5.0), neutrophils (9.92 × 10^9^ /L, RR 2.00–8.00), and platelets (487 × 10^9^ /L, RR 150–400). Computed tomography scanning of the neck was undertaken, which demonstrated subcutaneous abscesses in the left anterior and lateral neck tracking toward the carotid sheath and extending into the superior mediastinum with mediastinitis characterized by inflammatory phlegmon (Figure 1). A thrombosed left internal jugular, subclavian, and brachiocephalic vein was also visualized. Due to the deep extension of the abscesses, a CT chest was also conducted which demonstrated a pericardial effusion (Figure 2) and an area of consolidation in the medial aspect of the left upper lobe of the lung (Figure 3).

The patient was taken to the operating theater for surgical exploration, washout, and debridement. Incisions were made along the anterior border of the left sternocleidomastoid muscle which revealed two necrotic cavities with bloody serous fluid within. The cavities were washed with saline, and a Penrose drain was left in situ. A decision was made to explore the mediastinum using a left parasternal mediastinotomy approach. The Chamberlain procedure was conducted through the 2nd intercostal space with difficulty due to indurated and inflamed mediastinal tissue adherent to the pericardium and anterior chest wall. One hundred milliliters of hemorrhagic fluid was drained from the left pleura. The pericardium was opened close to the pulmonary artery with a small incision. Three hundred milliliters of serous fluid was drained with inflamed pericardium noted. Two silicone drains were placed in the left pleura before the thoracotomy wound was then closed in layers. The patient was successfully extubated post‐surgery and supported with high‐flow nasal prongs. The high‐flow oxygen was weaned to room air over the course of one day. The patient was uneventfully stepped down from intensive care one day post procedure.

**FIGURE 4:**
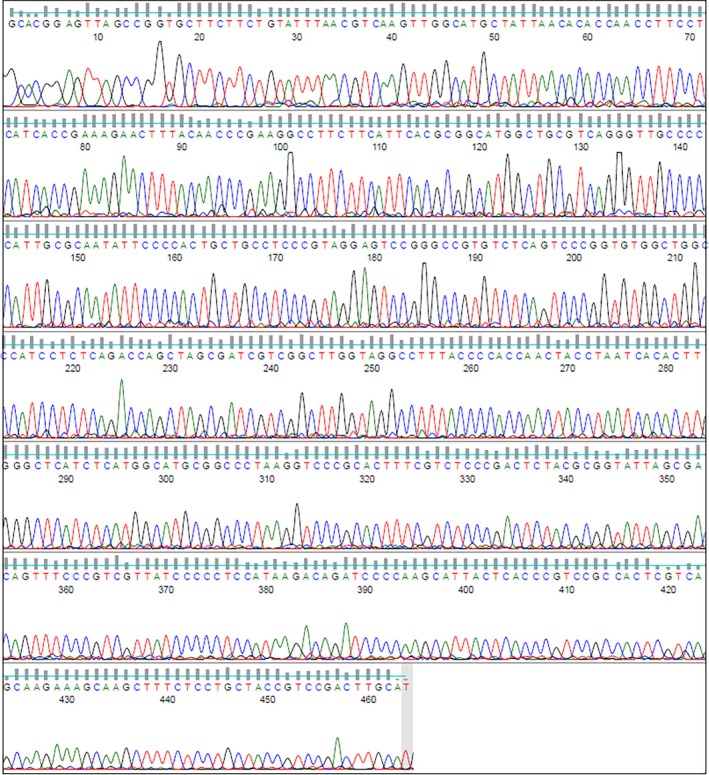
Chromatogram from 16 s RNA sequencing.

Intraoperative samples from the neck abscess, mediastinum and pericardium were sent for cytologic and microbiological testing. No organisms were seen on Gram's stain in all samples, although polymorphonuclear leukocytes were observed. Specimens that were incubated for five days in both aerobic and anaerobic conditions did not reveal any pathogens. Pericardial fluid cytological analysis revealed mesothelial cells mostly showing reactive atypia. To assist in identifying a microbiological cause, 16S rRNA sequencing was conducted on neck abscess tissue. 16S rRNA gene primer sets were applied to DNA extracts and amplified through polymerase chain reaction. Following checks for inhibition, the resultant amplicon was then sequenced by Sanger sequencing. This sequence (Figure 4) showed a 99% identification of *A. actinomycetemcomitans* using BLASTNR, FASTA, EZ Biocloud, and SeqMatch databases.

## 4 Differential Diagnosis and Treatment

In this case, infection was thought to be the most likely diagnosis given the acute presentation, history of purulent discharge, and systemic symptoms. Given the radiographic evidence of mediastinitis, the invasive extension of a neck infection was suspected. The formation of abscesses in the neck raised the possibility of *Staphylococcus aureus Streptococcus* spp. Over 50% of deep neck abscesses are polymicrobial, with both aerobes and anaerobes in most cases [1]. The internal jugular vein thrombosis raised the possibility of *Fusobacterium necrophorum* which is known to cause Lemierre's syndrome [3]. Other potential pathogenic oral anaerobes include *Bacteroides* spp., *Prevotella sp, Parvimonas micra* and *Peptostreptococcus* spp. [4, 5]. *Burkholderia pseudomallei* was considered a potential cause as the patient presented in Northern Australia, where melioidosis is seen during the wet season. Detection of *A. actinomycetemcomitans* by 16 s rRNA sequencing suggested that the infection was of periodontal origin. Subsequent dental orthopantomogram showed marked periodontal disease, supporting this hypothesis. Noninfectious causes such as malignancy and inflammatory syndromes were considered as part of the differential diagnosis.

Surgical intervention provided both diagnostic and therapeutic effects. Necrotic and purulent material was washed out, and drains were left in situ to facilitate ongoing drainage. The Penrose drain in the neck was removed one day following the procedure. The silicone drains in the left pleura drained a total cumulative output of 1.7 L over the course of twelve days and were removed when output had ceased. The patient experienced no surgical complications and was tolerating a full diet one day after his procedure. Further surgical debridement was not required. Upon initial presentation, the patient was commenced on intravenous meropenem 1 g three times a day for antimicrobial coverage which included the initially suspected *Burkholderia pseudomallei*. After two weeks of empirical therapy, diagnosis using 16S rRNA sequencing was obtained allowing the switch to intravenous ceftriaxone 2 g daily. Since the diagnosis was made using molecular methods, phenotypic antimicrobial susceptibility testing could not be conducted. Ceftriaxone was selected for targeted therapy as *A. actinomycetemcomitans* is reliably susceptible [6, 7]. Intravenous antibiotics were continued for a total of four weeks after which the patient was switched to oral amoxicillin‐clavulanate 875/125 mg twice daily. The patient continued oral therapy for two more weeks to complete a total of 6 weeks of antibiotic therapy post‐surgery. The patient was also started on warfarin for the deep vein thromboses.

## 5 Conclusions and Results

The patient remained an inpatient throughout the four‐week duration of his intravenous therapy. No rehabilitation was required. Post‐discharge, he was followed up in the outpatient department at three months, 6 months and 1 year post admission. In all reviews, the patient reported improvement and resolution of his clinical symptoms and good healing of his surgical wounds. Interval computed tomography of the neck and chest was repeated at six months and one year post admission, which demonstrated resolution of the mediastinitis and no inflammatory or neck soft tissue changes. Furthermore, his C‐reactive protein had normalized. The patient was advised to seek dental treatment due to the concern of a periodontal source and had several teeth extracted after review.

## 6 Discussion

*A. actinomycetemcomitans* was first described in 1912 where it was isolated with *Actinomyces* in cutaneous actinomycotic lesions, which is now a well‐known association [2]. It is also a known cause of juvenile and adult periodontitis, and its virulence appears to be determined by several virulence factors, such as the production of leukotoxins [8, 9]. Extra‐oral infections are rare. Most well‐known is its inclusion in the HACEK group of organisms, which are recognized as causes of infective endocarditis [10]. Bacteremia is strongly associated with endocarditis with a positive predictive value of 100% [11, 12]. It furthermore has been implicated in osteomyelitis, septic arthritis and brain abscess [13, 14, 15]. Recent case reports have highlighted its ability to cause a pneumonia that mimics pulmonary malignancy and invades the chest wall. The presence of periodontal disease appears to be a common risk factor among these cases. Hypothesized routes of invasion in these cases include aspiration followed by direct invasion or hematogenous spread [16, 17, 18]. Mediastinal involvement has been described in previous case reports as a mediastinal mass, sometimes with *Actinomyces* coinfection [19, 20, 21]. Invasion into the underlying vasculature has not been described. To our knowledge, a case of deep neck infection with invasion into the carotid sheath, mediastinal structures and provoked deep vein thrombosis has not been previously reported. We hypothesize that the infection was a result of oral infection followed by invasion into the deep spaces of the neck, the carotid sheath, and upper mediastinum. An alternate means of invasion would be aspiration leading to upper lobe pneumonia and then direct invasion into the neck and mediastinum. The recommended duration of treatment for non‐endocarditis, non‐oral infections is unclear. Reviews have described durations of 4 weeks to 1 year, depending on case severity as being successful [14, 18]. In our case, a 6‐week duration of antibiotics provided good clinical resolution and no relapse 1 year after treatment.

## Author Contributions

**Joshua Sia:** conceptualization, data curation, investigation, project administration, writing – original draft, writing – review and editing. **Carly Hughes:** conceptualization, supervision, writing – review and editing.

## Funding

No funding from internal or external sources was received in producing this manuscript.

## Consent

Written informed consent was obtained from the patient to publish this report in accordance with the journal's patient consent policy.

## Conflicts of Interest

The authors declare no conflicts of interest.

## Data Availability

The data that support the findings of this study are available on request from the corresponding author. The data are not publicly available due to privacy or ethical restrictions.
